# The effect of norepinephrine on common carotid artery blood flow in septic shock patients

**DOI:** 10.1038/s41598-021-96082-4

**Published:** 2021-08-18

**Authors:** Seok Goo Kim, Ik Joon Jo, Soo Yeon Kang, Jonghoon Yoo, Guntak Lee, Jong Eun Park, Taerim Kim, Sung Yeon Hwang, Won Chul Cha, Tae Gun Shin, Heewon Han, Hee Yoon

**Affiliations:** 1 Department of Emergency Medicine, Samsung Medical Center, Sungkyunkwan University School of Medicine, 115 Irwon-ro, Gangnam-gu, Seoul, 06355 Republic of Korea; 2 Department of Emergency Medicine, Dongsuwon General Hospital, Suwon-si, Gyeonggi-do Republic of Korea; 3 Department of Emergency Medicine, Keimyung University Dongsan Hospital, Daegu, South Korea; 4 Statistics and Data Center, Research Institute for Future Medicine, Samsung Medical Center, Seoul, South Korea

**Keywords:** Medical research, Cardiology, Infectious diseases, Medical imaging, Drug therapy

## Abstract

This study was designed to evaluate the hemodynamic effect of norepinephrine (NE) on the peak systolic velocity (PSV), diameter, and blood flow of the common carotid artery (CCA) using the point-of-care ultrasound (POCUS) in patients with septic shock. The study involved patients above 18 years old with septic shock. Arterial monitoring, carotid ultrasonography, and transthoracic echocardiography were performed before NE administration (T_0_). When the mean arterial pressure exceeded 65 mmHg after NE administration (T_1_), the measurement was repeated. Twenty-four patients (median age 67 [interquartile range: 54–77] years; 42% female) with septic shock were examined in this study. Before (T_0_) and after (T_1_) NE administration, the PSV (mean, standard deviation [SD]) changed from 85.3 (21.1) cm/s to 83.5 (23.5) cm/s (p = 0.417); this change was not significant. However, the diameter and blood flow of the CCA increased significantly from 0.6 (0.09) cm and 0.75 (0.27) L/min to 0.66 (0.09) cm and 0.85 (0.27) L/min, respectively (p < 0.001). The diameter of the left ventricular outflow tract (LVOT) remained unchanged, but the velocity time integral of the LVOT increased significantly from 21.7 (4.39) cm to 23.6 (5.14) cm. There was no significant correlation between changes in blood flow of the CCA and changes in cardiac output (coefficient −0.365, p = 0.079). In conclusion, NE increased the diameter and blood flow of the CCA significantly, without changing the PSV in patients with septic shock.

## Introduction

The incidence of septic shock, defined as sepsis with concurrent hypotension requiring vasopressor therapy, have been increased worldwide^1–4^. Norepinephrine (NE) is a first-line agent for the correction of hypotension, and its early administration is recommended for patients with septic shock. NE has α- and β1-adrenergic properties; therefore, it causes an increase in the mean arterial pressure (MAP), and small increases (10–15%) in the stroke volume and cardiac output (CO)^5–9^. Assessment of the hemodynamic status of patients with shock receiving NE is essential for an appropriate management of these patients. In recent decades, invasive hemodynamic monitoring has been replaced by the use of a non-invasive monitoring device or bedside ultrasonography^10^. However, using comprehensive echography proficiently to measure the cardiac output (CO) in emergency settings is not easy for physicians.

Carotid measurement is a much simpler technique, which can be performed with more accessibility on unstable patients with limited change of position. The blood flow of the common carotid artery (CCA) has been known to represent CO indirectly, showing moderate agreement with echocardiography^11^. Blood flow changes of the CCA by preload challenges in shock patient had already been reported in several^12–15^. A recent study by Marik et al. showed that the CCA blood flow rate increased by 60% after passive leg elevation, and predicted the volume response with 94% sensitivity and 86% specificity^16^. However, the hemodynamic effects of NE on the CCA blood flow in patients with septic shock have not been investigated. In addition, the correlation between CCA blood flow changes using the point-of-care ultrasound (POCUS) and CO changes using echocardiography according to NE administration is unclear.

Therefore, the aim of this study was to evaluate the hemodynamic effect of NE on the peak systolic velocity (PSV), diameter, and blood flow of the CCA using the POCUS in patients with septic shock. In addition, we investigated whether CCA measurements can be used as a method compared to echocardiography in evaluating the response to NE in patients with septic shock.

## Materias and methods

### Study design and setting

This prospective observational study was conducted from December 2019 to February 2020 at the emergency department of an academic tertiary hospital in an urban area. The study was conducted in accordance with the Declaration of Helsinki. The study was approved by the institutional review board committee of Samsung Medical Center (IRB File Number: 2019-09-102-003) and clinical research information service (CRIS, Approval Number: KCT0004559). Informed consent was provided by all patients or guardians if the patient was unconscious, disoriented, above 65 years old, or had dementia.

### Selection of participants

We screened patients aged over 18 years with identified or developed sepsis-induced hypotension after visiting the emergency department (systolic blood pressure < 90 mmHg or MAP < 70 mmHg with a heart rate [HR] > 100 rates/min). Patients with persistent hypotension requiring vasopressor use for the maintenance of an MAP above 65 mmHg despite adequate fluid resuscitation (> 2000 mL or 30 mL/kg within 3 h) were enrolled^17–20^.

Pregnant patients, trauma patients, patients with a do-not-resuscitate status, and patients with cardiac arrest were excluded. Additionally, patients who were receiving or had received a vasopressor within 24 h prior to the study were excluded. Patients with a CCA stenosis degree of ≥ 50% on carotid ultrasonography (PSV > 150 cm/s with post stenotic turbulence) and those with a medical history of CCA stenosis were excluded^21, 22^. Patients who were unable to undergo follow-up ultrasonography and who did not give consent to the use of their data were also excluded.

### Methods and measurements

#### Study protocol

**Figure 1:**
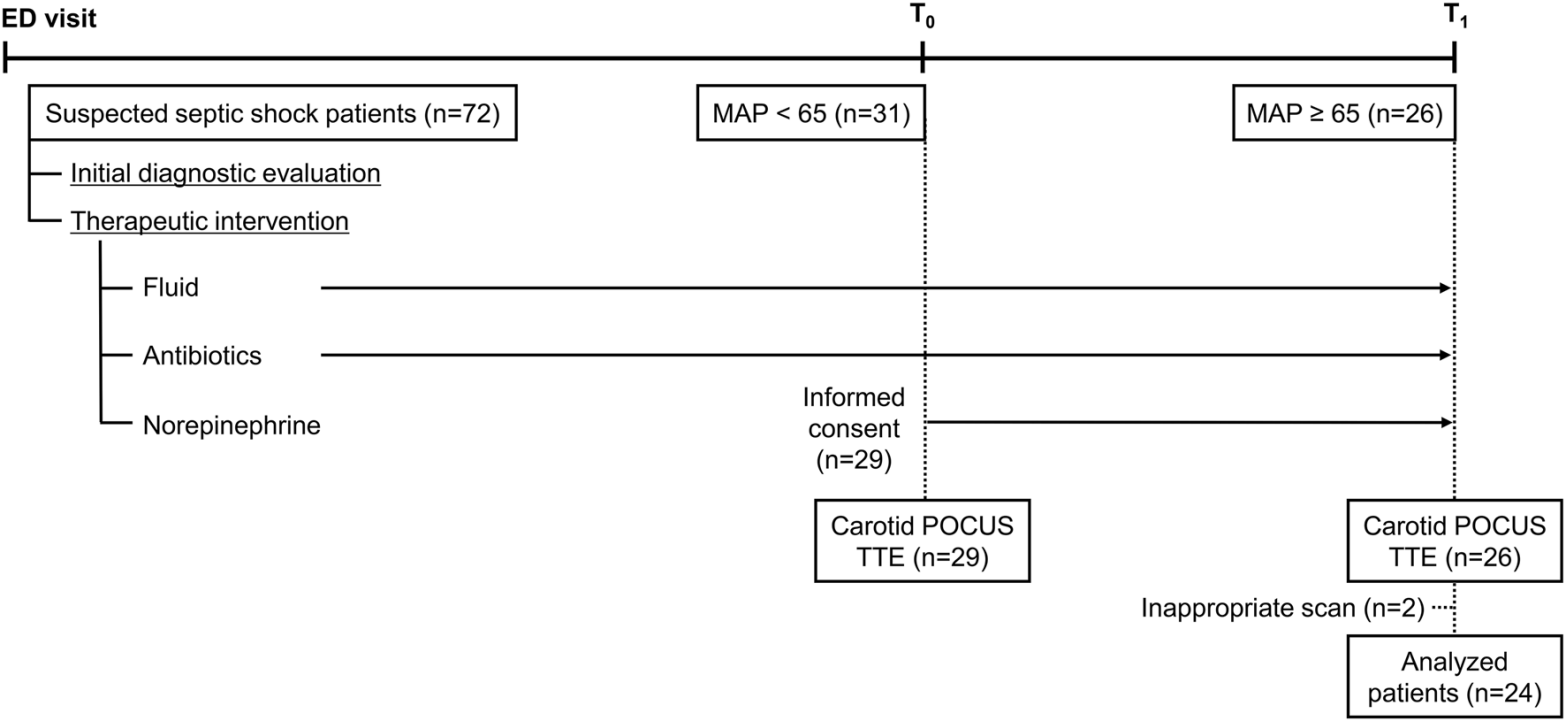
Study timeline. ED, emergency department; T_0,_ time before initiation of norepinephrine; T_1_, time after initiation of norepinephrine; MAP, mean arterial pressure; POCUS, point-of-care ultrasound; TTE, transthoracic echocardiography.

All patients suspected of septic shock received initial diagnostic evaluations: these included laboratory and imaging work-up, concurrently with therapeutic interventions such as fluid administration and antibiotic therapy, according to Survival Sepsis Campaign guidelines^19^. They underwent close monitoring via the arterial line at the radial artery, and POCUS was initially performed for cause assessment and patient management. Patients who required NE for the maintenance of an MAP above 65 mmHg even after initial resuscitative treatment were enrolled (Fig. 1). The PSV and CCA diameter was measured using carotid POCUS, and the velocity time integral (VTI) and diameter of the left ventricular outflow tract (LVOT) was measured using transthoracic echocardiography (TTE) before NE administration (T_0_). When the MAP reached a level above 65 mmHg for 10 min after NE administration (T_1_), a re-measurement was performed. Additionally, the patients’ demographic, laboratory, and hemodynamic data were collected through arterial line monitoring at T_0_ and T_1_.

#### Ultrasonography

All sonographic examinations were performed by a single operator experienced in emergency medicine POCUS, with convenient sampling according to the protocol. Bedside ultrasonography was performed with vivid ™ S70 (GE healthcare, Horten, Norway/program version: 202). Two-dimensional (2D) image mode and Pulsed Wave (PW) Doppler image mode with linear probe (11L) on vascular preset were used for CCA identification and estimation. TTE was performed with a cardiac probe (M5Sc) for the measurement of the diameter and VTI of the LVOT on cardiac preset.

Ultrasonography was performed within 5 min, and the obtained images were saved as video files, and then reviewed by two attending emergency physicians who served as ultrasonography instructors. When the measurements were reviewed using six indicators, a score of ≥ 4 was considered clinically interpretable and included in the analysis; otherwise, it was excluded (Supplemental Table 1A)^23–25^. The carotid and cardiac measurements were repeated three times if possible, and then averaged for the minimization of random errors.

#### Carotid measurement

To increase the accuracy of the repeat measurements, a pillow was used to ensure uniformity in the patients’ neck positions. We marked the center and edge of the probe on the patient’s neck for easy re-measurement at the same location (Supplemental Fig. 1)^23^. The CCA was identified in the short-axis and the long-axis view. PW Doppler ultrasonography was performed at the center of the vessel with angle correction settings of 60° at 1–2 cm below the carotid bulb. The additional color Doppler mode was used for post-stenotic turbulence distinction. The CCA diameter was measured from opposite points of the vessel’s intimal wall. The PSV and VTI were determined automatically from the sample obtained from the center of the artery using PW Doppler (Supplemental Fig. 1)^23, 24^. The CCA blood flow per minute was calculated using the equation: π × (CCA diameter)^2^/4 × CCA VTI × HR. This parameter was measured both before and after NE administration for the determination of CCA blood flow changes.

#### Cardiac measurement

The patients were maintained in a supine position for image acquisition in the parasternal and apical windows. The edge of the probe on the patient’s chest was marked for easy re-measurements at the same location. The diameter of the LVOT was considered as the inner distance between the bases of the aortic valve cusp during systole, as observed from the parasternal long-axis view. PW Doppler samples were then obtained at the center of the LVOT from the apical view, with the aim of obtaining a Doppler signal to aortic blood flow angle close to 0°^25^. LV stroke volume was estimated after obtaining the VTI of the PW measurement at the LVOT. The CO per minute was calculated using the equation π × (LVOT diameter)^2^/4 × LVOT VTI × HR.

#### Outcomes

The primary outcome was the PSV change in the CCA observed before NE administration (T_0_) and after the achievement of an MAP above 65 mmHg after NE administration (T_1_). The secondary outcome was the CCA diameter and blood flow change between T_0_ and T_1_. In addition, we measured the diameter, VTI of the LVOT, and CO changes by TTE according to the NE administration status. We also assessed the correlation between CCA flow changes via POCUS and CO changes via TTE. We analyzed the factors affecting the CCA blood flow change according to the NE status. For reliability evaluation, the intra-operator repeatability value was also calculated.

### Statistical analyses

The sample size was calculated relative to the primary outcome achievement; we assumed an α value of 0.05 for two-sided hypothesis testing and a β error of 0.20 (power = 80%). A 15% increase in the CCA PSV following NE administration was considered clinically significant^7–9^. We used an inequality test for paired means based on a previous literature wherein the PSV (93.6 ± 20.7) in the CCA was used^26^. We assumed a drop-out rate of 10%. A total of 22 patients was deemed required.

Standard descriptive statistics are used to present all data. Continuous variables are presented as mean (standard deviation [SD]) or median (interquartile range [IQR]). Categorical data are presented as numbers with percentages. Paired t-tests or Wilcoxon signed-rank tests were used for mean comparisons between the two groups. Univariate and multivariate regression analyses were performed to determine the factors affecting CCA blood flow changes. Variables for which analyses resulted in p values lower than 0.2 were incorporated into the multivariate regression analysis. The relationship between CCA flow changes and CO changes was assessed using Pearson’s correlation analysis. Intra-operator repeatability was evaluated by comparing the three variable measurements for the calculation of intra-class correlation coefficients (ICCs) with their 95% confidence intervals (CIs). All statistical analyses were performed using STATA ver. 15.0 software (STATA Corporation, College Station, TX).

## Results

### Study patients and baseline characteristics

A total of 72 patients were screened, and there were 31 patients with persistent hypotension requiring vasopressor use after sufficient fluid resuscitation. Two of these patients refused to provide consent, and three had incomplete follow-up ultrasonography data: they were excluded. Two patients with ultrasonography images considered as inappropriate during the image review were also excluded. Finally, 24 patients were included in the analysis (Fig. 1). Ten of the patients (42%) were women. The median (IQR) patient age was 67 (54–77) years. The most commonly reported cause of septic shock was urinary tract infection, followed by pneumonia and cholangiohepatitis. At T_0,_ 1.9 ± 0.69 L of fluid was administered. The mean (SD) NE dose was 0.11 (0.05) mcg/kg/min (Table 1).

**Table 1:** Baseline characteristics of patients.

	N = 24
Age (years)	67 (54–77)
Female (n, %)	10 (42)
**Co-morbidities (n, %)**	
Hypertension	12 (50)
Diabetes mellitus	5 (21)
Ischemic heart disease	1 (4)
Pulmonary disease	2 (8)
Cancer	19 (79)
Chronic renal disease	1 (4)
Chronic liver disease	3 (13)
**Septic shock causes (n, %)**	
Urinary tract infection	7 (29)
Pneumonia	6 (25)
Cholangiohepatitis	6 (25)
Neutropenia	4 (17)
Colitis	4 (17)
combined with hypovolemia	14 (58)
Carotid artery calcification (n, %)	6 (25)
**Amount of fluid administered**	
At T_0_ (L)	1.9 (0.69)
At T_1_ (L)	2.6 (0.76)
**Norepinephrine**	
Initiation time from ED visit (min)	222 (110)
Dose (mcg/kg/min)	0.1 (0.05)
Dose (mcg/kg)	8.5 (5.3 – 15.9)

T_0,_ time before initiation of norepinephrine; T_1,_ time after initiation of norepinephrine; ED, emergency department.

Data are presented as mean (standard deviation) and median (interquartile range).

### Hemodynamic and laboratory variables

There was no significant change in HR (mean, SD) from T_0_ to T_1_— from 96 (18) to 94 (19) beats/min. The median blood lactate level (IQR) at T_1_ was 1.8 (1.3–2.8) mmol/L, which was significantly lower than 2.85 (2.5–5.5) mmol/L at T_0_ (Supplemental Table 2).

### Outcome analysis

**Figure 2:**
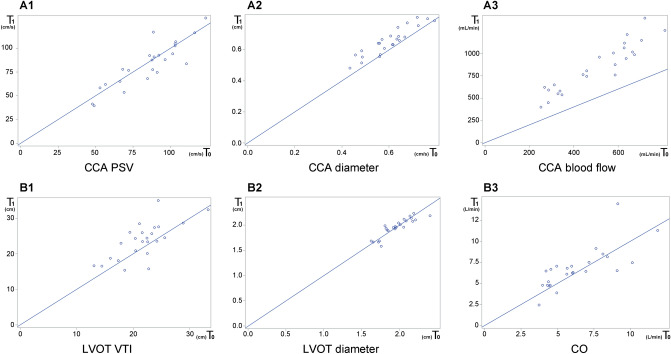
Scatter plot of carotid POCUS and TTE before (T_0_) and after (T_1_) restoration of a MAP ≥ 65 mmHg with norepinephrine in the whole population (n = 24) : A1 (CCA PSV), A2 (CCA diameter), A3 (CCA blood flow), B1 (LVOT VTI), B2 (LVOT diameter), B3 (CO). POCUS, point-of-care ultrasound; TTE, transthoracic echocardiography; MAP, mean arterial pressure; CCA, common carotid artery; PSV, peak systolic velocity; LVOT, left ventricular outflow tract; VTI, velocity time integral; CO, cardiac output The lines in each graph represent the same unit values on the X (T_0_) and Y (T_1_) axes.

Before (T_0_) and after (T_1_) NE administration, the mean (SD) of the PSV and CCA diameter were 85.3 (21.1) cm/s, 0.6 (0.09) cm and 83.5 (23.5) cm/s, 0.66 (0.09) cm, respectively. The CCA PSV remained unchanged, but the CCA diameter increased significantly. The CCA blood flow also increased significantly from 0.75 (0.27) L/min to 0.85 (0.27) L/min after NE administration (p < 0.001). The change was approximately 16 (SD 20) % (Table 2 and Fig. 2).

**Table 2:** Carotid variables by POCUS and cardiac variables by TTE before (T_0_) and after (T_1_) initiation of norepinephrine.

	T_0_	T_1_	p-value
MAP (mmHg)	56 (4)	71 (6)	< 0.001
**Carotid variables**			
PSV (cm/s)	85.3 (21.1)	83.5 (23.5)	0.417
VTI (cm)	43.6 (11.5)	42.4 (11.5)	0.206
CCA Diameter (cm)	0.6 (0.09)	0.66 (0.09)	< 0.001
CCA blood flow (L/min)	0.75 (0.27)	0.85 (0.27)	< 0.001
**Cardiac variables**			
VTI (cm)	21.7 (4.39)	23.6 (5.14)	< 0.018
LVOT Diameter (cm)	1.96 (0.2)	1.95 (0.18)	0.693
CO (L/min) (median, IQR)	5.8 (4.5–7.9)	6.5 (5.0–7.5)	0.170
IVC CI (n = 22)	0.41 (0.17)	0.37 (0.19)	0.194

POCUS, point-of-care ultrasound; MAP, mean arterial pressure; TTE, transthoracic echocardiography; PSV, peak systolic velocity; VTI, velocity time integral; CCA, common carotid artery; LVOT, left ventricular outflow tract; CO, cardiac output; IVC CI, inferior vena cava collapsibility index.

Data are presented as mean (standard deviation) and median (IQR, interquartile range).

Before (T_0_) and after (T_1_) NE administration, the VTI and diameter of the LVOT were 21.7 (4.39) cm/s, 1.96 (0.2) cm and 23.6 (5.14) cm/s, 1.95 (0.18) cm, respectively (Table 2). In contrast to the carotid measurement values, the diameter of the LVOT from T_0_ to T_1_ remained unchanged, but the VTI of the LVOT increased significantly.

Although the carotid blood flow increased in all the patients after NE administration, TTE CO decreased by 1.281 (0.983) L/min in seven patients at T1. The carotid blood flow changes in the seven patients with decreased CO was 0.17 L/min (SD 0.07), which was significantly greater than the change of 0.06 L/min (SD 0.11) in 17 patients with increased CO at T1. (p = 0.023) (Table 3). There was no significant correlation between changes in blood flow of CCA using POCUS and changes in CO using echocardiography (coefficient −0.003, p = 0.079).

**Table 3:** Comparison of carotid blood flow changes according to CO changes between T_0_ and T_1_.

Common carotid blood flow changes (L/min)	*N*	Mean	SD	95% CI		*T*	*P* value
Positive CO change [+ 1.166 (1.28) L/min]	17	0.06	0.11	0.01	0.12	−2.44	0.023
Negative CO change [− 1.281 (0.983) L/min]	7	0.17	0.07	0.1	0.24		
Total	24	0.1	0.11	0.05	0.14		

CO, cardiac output; SD, standard deviation; CI, confidence interval.

A multivariate regression analysis to evaluate the factors influencing CCA blood flow changes was performed after adjusting for C-reactive protein (CRP), NE dose, MAP (T_0_), HR (T_0_), and pH (T_0_). CCA blood flow change (L/min) decreased in association with high MAP values at T_0_ (coefficient −0.016, p = 0.023) and increased in association with high HR values at T_0_ (coefficient + 0.005, p = 0.003) (Supplemental Tables 3, 4).

The intra-operator repeatability tests of the carotid and cardiac measurements showed ICCs of 80–94% (Supplemental Table 5). The ICC of the carotid measurements (92–94%) performed using POCUS was excellent and superior to that of the cardiac measurement (80–88%) conducted using TTE.

## Discussion

POCUS is increasingly used in the management of critically ill patients as a noninvasive method for hemodynamic monitoring and CO measurement^27, 28^. Several previous studies have examined the correlation between CA blood flow and CO^13, 16^. Gassner M. et al. showed that the reliability of carotid POCUS, compared to that of invasive measurements such as pulmonary artery catheterization or arterial waveform pulse contour analysis, was 74–84%, showing more than a substantial degree of agreement^12^. In the study by Peng et al., the overall ICC between the carotid and echocardiographic CO was 0.537, but a subgroup analysis of 14 patients with septic shock showed a weak correlation with an ICC of 0.241. In addition, vasopressor use was not considered^11^. In this study, the mean value of CCA blood flow increased significantly by approximately 16 (SD 20) % when MAP increased after NE administration (Table 2). However, there was no significant correlation between changes in CCA blood flow measured by POCUS and changes in CO measured by TTE. This may be due to the changes in blood distribution among vital organs and autoregulation of cerebral blood flow in patients with septic shock, as seen in other animal studies^29, 30^. Therefore, carotid POCUS can be used as a tool to assess the increase of MAP to NE, but it may not be reliable to use as an alternative CO assessment method compared to echocardiography in patients with septic shock.

As NE exerts α- and β1-adrenergic agonist effects^8^, we expected that the CCA diameter would decrease by vasoconstriction after the administration of NE, and the PSV would increase, resulting in an increase in the rate of blood flow in patients with septic shock. Conversely, in this study, the CCA diameter increased significantly without meaningful changes in the PSV after NE administration. When vasoactive drugs are administered, the actual change in the diameter of the vessel may reflect the net effect of passive dilatory forces and active smooth muscle contraction. A previous study on phenylephrine (α1 selective adrenergic agonist) conducted by Bonyhay et al. also showed similar results in six volunteers with normal blood pressure values^31^. Our findings suggest that when NE is administered to patients with septic shock, the passive dilatory forces resulting from the increased carotid blood flow are likely to dominate the active tension developed by smooth muscle contraction in the CCA. Also, since CCA blood flow reflects intracranial blood flow, this is thought to be related to cerebral autoregulation to maintain constant blood flow in the brain of patients with septic shock^32^.

The CO increases that occur following NE use are associated with an increase in the total end-diastolic volume, which is an indicator of cardiac preload^33, 34^. However, Hamzaou et al. suggested that NE may increase cardiac contractility through both an indirect α-adrenergic effect by improving coronary perfusion according to the increase in diastolic arterial pressure and a direct effect on cardiomyocytes via β1-adrenergic stimulation, mainly in the early phase of septic shock^5, 6^. In this study, the CO value was increased by approximately 9 (SD 24) % from 5.83 (IQR 4.46–7.89) L/min to 6.50 (IQR 4.99–7.49) L/min after NE application. Whereas, in seven patients, the CO decreased at T_1_. This may be related to a negative effect of NE on cardiac function through an increase in left ventricular afterload^35^. The carotid blood flow change in the seven patients with decreased CO was 0.17 (SD 0.07) L, which was significantly greater than the change in patients with increased CO at T_1_ by about three-fold. (p = 0.023) (Table 3). Therefore, NE can increase MAP through α- and β1-adrenergic properties, but in this study it was shown that the α-adrenergic effect of increasing the depressed vascular tone was more dominant in patients with septic shock, especially those sensitive to afterload.

We analyzed several factors that affect CCA blood flow changes, according to the NE status, using univariate and multivariate analyses (Supplemental Table 3, 4). MAP and HR at T_0_ were significantly correlated with CCA blood flow changes in the multivariate regression analysis after adjustment for PaCO2, pH, NE dose, CRP, and hydration volumes. That is, in the evaluation of the carotid blood flow response to NE, the change in the CCA blood flow is likely to increase when the initial MAP is low (coefficient −0.016, p = 0.023) or the initial HR is high (coefficient + 0.005, p = 0.003). However, these findings are thought to be related to the study design, and its generalizability is limited due to the small sample size.

The reliability of Doppler velocity measurements is severely affected by their intrinsic operator and machine dependency, with the beam-to-flow angle exerting the strongest effect^36, 37^. In this study, the ICC of the CCA measurement ranged between 92 (95% CI 86–96)% in the CCA diameter and 94 (95% CI 88–97)% in the CCA PSV, indicating that carotid measurements performed using POCUS have an excellent reliability and are higher than the cardiac measurements (80–88%) (Supplemental Table 5). Since most emergency physicians are not professional sonographers, they often find it difficult to measure the level of CO by flow velocity through the LVOT, especially when dealing with suboptimal cardiac windows due to positioning difficulties in patients with shock. However, carotid POCUS is easier to approach superficially and insonate at the bedside than accessing the LVOT. In addition, carotid POCUS can be serially repeated in the same patient, allowing clinicians to adequately assess the response to volume-loading maneuvers or NE application. It can be particularly helpful in the monitoring of patients with septic shock in time-critical clinical situations, in resource-limited environments, or those with no other available options. Therefore, further studies are needed to assess patients with septic shock in several settings using carotid POCUS.

## Limitations

This study has several limitations. First, although it had a prospective design, it was limited by the observational nature of the research and the lack of a control group. In addition, it was performed in a single center and involved few patients; as such, the results not be generalizable to other settings. Hence, further studies in other settings are needed.

Second, the patients’ comorbidities and drug use history, including use of antihypertensive drugs, may have affected the effectiveness of NE. In addition, since many patients had a hypovolemic component and the volume was administered between T_0_ and T_1_; i.e., during NE administration, these may have influenced preload dependence to a certain extent, in addition to the NE effect. Furthermore, a relatively small amount of NE (0.1 mcg/kg/min, mean) was administered in this study, and the effect on patients with refractory septic shock who required a high dose of NE could not be evaluated. Therefore, additional studies are needed on the effect of hypovolemia and NE dose on carotid and cardiac measurements following NE administration.

Third, this study design did not include blinding the operator. Also, ultrasonography is operator-dependent. Even if PW Doppler is performed at the center of the vessel with an angle correction of 60°, a small difference in the angle and degree parallel to the vessel can cause the measurements to differ. Moreover, the arterial blood pressure of some patients changed dynamically, even during ultrasonography. Therefore, there may be some discrepancies between the MAP when measuring the PSV and the actual value. In addition, although we took the measurements at the same location, there may be differences in the values according to the change in the patient’s position and measurement site.

Lastly, we measured both carotid and cardiac values non-invasively using ultrasonography, and none of these could be compared to exact baseline values when assessing their relationship. In addition, we could not provide other cardiac measurements to evaluate the cardiac functions except for CO measurements. Therefore, it was not possible to explain in more detail the changes of cardiac functions after NE administration. Moreover, since we did not measure outcomes serially after NE administration, changes such as compensation or desensitization of the effects of NE over time are not known.

## Conclusions

In summary, NE use leads to increases in the CCA diameter and blood flow rate without significant changes in the CCA PSV in patients with septic shock; these were observed using carotid POCUS. The measurements of CCA blood flow using carotid POCUS may have the potential of being used in the assessment of MAP increase in response to NE in patients with septic shock.

## Supplementary Information

Supplementary Information 1.

Supplementary Information 2.

Supplementary Information 3.

Supplementary Information 4.

Supplementary Information 5.

Supplementary Information 6.
