# Supplementary material for: The effect of norepinephrine on common carotid artery blood flow in septic shock patients
Source: Sci Rep. 2021 Aug 18;11:16763. doi: 10.1038/s41598-021-96082-4 (PMC8373863; doi:10.1038/s41598-021-96082-4)
Supplement: Supplementary file 1 — Supplementary Information 1. [file 41598_2021_96082_MOESM1_ESM.docx]

Supplemental Figure 1 Carotid artery measurements by the carotid POCUS


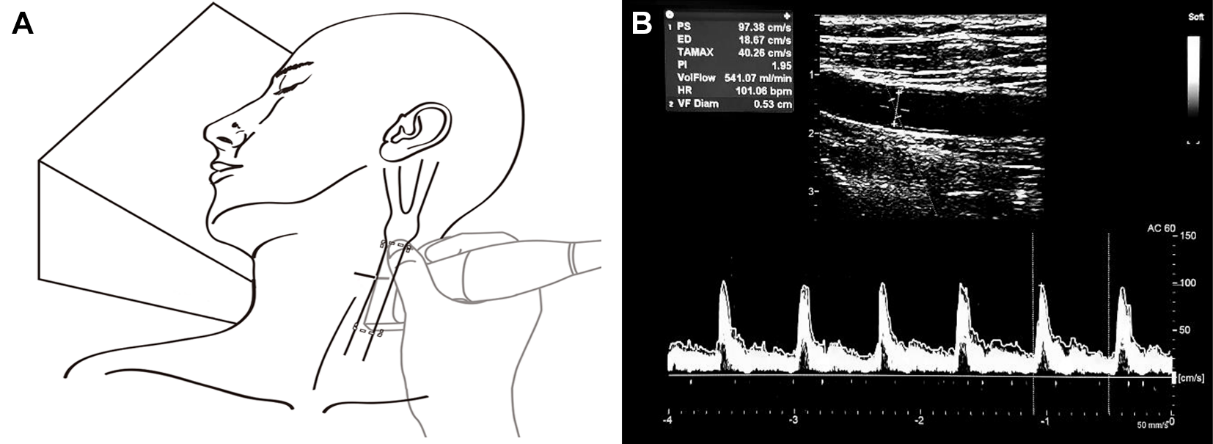


| **A.** Schematic patient position image for measuring common carotid artery | **B.** A 2-dimensional mode with Pulsed Wave Doppler ultrasonography image. |
| --- | --- |

POCUS, point-of-care ultrasoun
