# Supplementary material for: The effect of norepinephrine on common carotid artery blood flow in septic shock patients
Source: Sci Rep. 2021 Aug 18;11:16763. doi: 10.1038/s41598-021-96082-4 (PMC8373863; doi:10.1038/s41598-021-96082-4)
Supplement: Supplementary file 2 — Supplementary Information 2. [file 41598_2021_96082_MOESM2_ESM.docx]

**Supplemental Table 1. Criteria and results for evaluation of the adequacy of carotid and cardiac measurements**

**Supplemental Table 1A.** Criteria for evaluation of the adequacy of carotid and cardiac measurements

Total Score >= 4: clinically interpretable

Total Score <= 4: clinically uninterpretable

| Carotid POCUS Measurements | Score |
| --- | --- |
| Measurement of diameter obtained in systole | 1 |
| Diameter measured 1-2cm proximal to carotid bulb | 1 |
| Diameter measured from intima to intima | 1 |
| Doppler gate placed at the center of the CCA | 1 |
| Doppler gate parallel to CCA wall with angle correction of 60° | 1 |
| Well-defined spectral waveform obtained | 1 |
| Echocardiographic Measurements | Score |
| Measurement of diameter obtained in systole | 1 |
| Diameter measured within 0.5 to 1cm to the aortic annulus | 1 |
| Diameter measured from inner to inner | 1 |
| Doppler gate placed in the center of the LVOT at apical view | 1 |
| Doppler gates parallel to the LVOT at the proper angle (<20°) | 1 |
| Well-defined spectral waveform obtained | 1 |

CCA, common carotid artery; LVOT, left ventricular outflow tract

**Supplemental Table 1B.** Results of the evaluation of the adequacy of carotid and cardiac measurements

|  | Carotid measurement | Cardiac measurement |
| --- | --- | --- |
| Score (mean, SD) | 5.4 (0.7) | 5.1 (0.7) |

SD, standard deviation
