# Supplementary material for: The effect of norepinephrine on common carotid artery blood flow in septic shock patients
Source: Sci Rep. 2021 Aug 18;11:16763. doi: 10.1038/s41598-021-96082-4 (PMC8373863; doi:10.1038/s41598-021-96082-4)
Supplement: Supplementary file 3 — Supplementary Information 3. [file 41598_2021_96082_MOESM3_ESM.docx]

Supplemental Table 2. Hemodynamic and laboratory variables before (T_0_) and after (T_1_) initiation of norepinephrine (n=24)

|  | T_0_ | | T_1_ | p-value |
| --- | --- | --- | --- | --- |
| Hemodynamic variables | | | | |
| Heart rate (rate/min) | 96 (18) | | 94 (19) | 0.174 |
| SPO_2_ (%) | 98 (1.9) | | 94 (2.3) | 0.429 |
| Body temperature (°C) | 37.4 (0.8) | | 37.4 (0.6) | 0.721 |
| SAP (mmHg) | 84 (6) | | 109 (11) | <0.001 |
| DAP (mmHg) | 42 (6) | | 50 (12) | 0.002 |
| MAP (mmHg) | 56 (4) | | 71 (6) | <0.001 |
| Laboratory variables | | | | |
| Lactate (mmol/L) | | 2.85 (2.5 - 5.5) | 1.8 (1.3 – 2.8) | <0.001 |
| pH | 7.462 (0.449) | | 7.461 (0.043) | 0.908 |
| iCa (mg/dL) | 0.981 (0.753) | | 1.025 (0.093) | 0.013 |
| PaCO_2_ (mmHg) | 31 (5.1) | | 31.2 (5.1) | 0.71 |

SPO_2,_ saturation of percutaneous oxygen, SAP, systolic arterial pressure; MAP, mean arterial pressure; DAP, diastolic arterial pressure; iCa, ionized calcium; PaCO_2_, partial pressure of carbon dioxide in arterial blood
Data are presented mean (standard deviation) and median (interquartile ranges).
