# Supplementary material for: The effect of norepinephrine on common carotid artery blood flow in septic shock patients
Source: Sci Rep. 2021 Aug 18;11:16763. doi: 10.1038/s41598-021-96082-4 (PMC8373863; doi:10.1038/s41598-021-96082-4)
Supplement: Supplementary file 4 — Supplementary Information 4. [file 41598_2021_96082_MOESM4_ESM.docx]

**Supplemental Table 3.** Supplemental Table 4. Univariate regression analysis of factors affecting carotid blood flow changes (L/min)

| Variable | Coefficient | SE | p-value | 95% CI | |
| --- | --- | --- | --- | --- | --- |
| Age (year) | -0.00222 | 0.00191 | 0.259 | -0.00596 | 0.00152 |
| Female | 0.00113 | 0.04484 | 0.98 | -0.08676 | 0.08902 |
| Body weight (kg) | -0.00083 | 0.00183 | 0.656 | -0.00441 | 0.00276 |
| Pre-hydration volume (mL) | -0.0037 | 0.03228 | 0.909 | -0.06697 | 0.05957 |
| Post-hydration volume (mL) | -0.02768 | 0.04584 | 0.552 | -0.11752 | 0.06217 |
| Pre-Systolic atrial pressure (mmHg) | 0.00307 | 0.00377 | 0.424 | -0.00432 | 0.01046 |
| Pre-Diastolic atrial pressure (mmHg) | -0.00495 | 0.00391 | 0.219 | -0.01261 | 0.00271 |
| Pre-Mean atrial pressure (mmHg) | -0.0093 | 0.00508 | 0.081 | -0.01926 | 0.00066 |
| Pre-Heart rate (rate/min) | 0.00226 | 0.00113 | 0.058 | 0.00005 | 0.00447 |
| Pre-Body temperature (°C) | 0.02066 | 0.02713 | 0.455 | -0.03251 | 0.07383 |
| Pre-pH | -0.69927 | 0.48075 | 0.16 | -1.64154 | 0.243 |
| Pre-SPO_2_ (%) | 0.00657 | 0.01142 | 0.571 | -0.01581 | 0.02895 |
| Pre-PaCO_2_ (mmHg) | -0.00013 | 0.00037 | 0.733 | -0.00085 | 0.00059 |
| Pre-iCa_2_^+^ (mmol/L) | -0.15682 | 0.29818 | 0.604 | -0.74125 | 0.42761 |
| Pre-Lactate (mmol/L) | 0.01298 | 0.01409 | 0.367 | -0.01463 | 0.04059 |
| CRP (mg/dL) | -0.00234 | 0.00176 | 0.197 | -0.00579 | 0.00276 |
| Norepinephrine dose (mcg/kg/min) | 0.64455 | 0.42923 | 0.147 | -0.19674 | 1.48584 |
| Norepinephrine dose (mcg/min) | 0.00088 | 0.00242 | 0.721 | -0.00387 | 0.00562 |
| Shock cause | 0.00013 | 0.04566 | 0.998 | -0.08937 | 0.08962 |

SE, standard error, CI, confidence interval
SPO_2,_ saturation of percutaneous oxygen; PaCO_2_, partial pressure of carbon dioxide in arterial blood; iCa, ionized calcium; CRP, C-reactive protein
