# Supplementary material for: The effect of norepinephrine on common carotid artery blood flow in septic shock patients
Source: Sci Rep. 2021 Aug 18;11:16763. doi: 10.1038/s41598-021-96082-4 (PMC8373863; doi:10.1038/s41598-021-96082-4)
Supplement: Supplementary file 5 — Supplementary Information 5. [file 41598_2021_96082_MOESM5_ESM.docx]

**Supplemental Table 4.** Multivariate regress analysis of factors affecting carotid blood flow changes

| Common carotid blood flow change (L) | Coefficient | SE | *p*-value | 95 % CI | |
| --- | --- | --- | --- | --- | --- |
| Pre-Mean atrial pressure (mmHg) | -0.01587 | 0.00627 | 0.023 | -0.02815 | -0.00359 |
| Pre-Heart rate (rate/min) | 0.00451 | 0.00124 | 0.003 | 0.00208 | 0.00694 |
| Pre-pH | -0.08048 | 0.44657 | 0.859 | -0.95576 | 0.79479 |
| Pre-hydration volume (mL) | 0.00636 | 0.02747 | 0.82 | -0.04748 | 0.0602 |
| Post-hydration volume (mL) | 0.00615 | 0.05728 | 0.916 | -0.10612 | 0.11841 |
| Pre-PaCO_2_ (mmHg) | -0.0002 | 0.00031 | 0.526 | -0.00081 | 0.00041 |
| CRP (mg/dL) | -0.00282 | 0.0017 | 0.118 | -0.00615 | 0100051 |
| Norepinephrine dose (mcg/kg/min) | -0.1397 | 0.41519 | 0.86 | -0.95347 | 0.79479 |
| Constant | 1.18818 | 3.28216 | 0.722 | -0.00615 | 0.00051 |

SE, standard error, CI, confidence interval
SPO_2,_ saturation of percutaneous oxygen; PaCO_2_, partial pressure of carbon dioxide in arterial blood; iCa, ionized calcium; CRP, C-reactive protein
