# Supplementary material for: The effect of norepinephrine on common carotid artery blood flow in septic shock patients
Source: Sci Rep. 2021 Aug 18;11:16763. doi: 10.1038/s41598-021-96082-4 (PMC8373863; doi:10.1038/s41598-021-96082-4)
Supplement: Supplementary file 6 — Supplementary Information 6. [file 41598_2021_96082_MOESM6_ESM.docx]

Supplemental Table 5. Intra-class correlation coefficients of the carotid and cardiac measurements

|  | ICC | 95 % CI |
| --- | --- | --- |
| Cardiac LVOT VTI | 0.88 | 0.79 - 0.94 |
| Cardiac LVOT diameter | 0.8 | 0.66 - 0.9 |
| Common carotid artery PSV | 0.94 | 0.88 - 0.97 |
| Common carotid artery diameter | 0.92 | 0.86 - 0.96 |

ICC, interclass correlation coefficient; CI, confidence interval; LVOT, left ventricular outflow tract; VTI, velocity time integral
